# Long-term conservation tillage improves water use efficiency of wheat by optimizing root water uptake and evapotranspiration components in a semiarid region

**DOI:** 10.3389/fpls.2026.1845937

**Published:** 2026-05-19

**Authors:** Jing Xu, Ning Ding, Le Wang, Yuanhong Zhang, Haofeng Meng, Lingling Li

**Affiliations:** 1 State Key Laboratory of Aridland Crop Science, College of Agronomy, Gansu Agricultural University, Lanzhou, Gansu, China; 2 Potato Research Institute, Gansu Academy of Agricultural Sciences, Lanzhou, Gansu, China

**Keywords:** Mechanism, no tillage, stable oxygen isotope, straw returning, water use efficiency, wheat

## Abstract

Conservation tillage is a primary strategy in dryland farming systems, that can significantly improve water productivity in dryland crops. However, the mechanism of conservation tillage drives optimization of root water uptake and evapotranspiration (ET) components to enhance efficient water utilization is unclear. Therefore, a two-year field experiment was carried out with spring wheat based on a long-term conservation tillage experiment in a semiarid region of Northwestern China to determine root water uptake, quantify evaporation and transpiration, and assess their relationship with yield and water use efficiency (WUE) under different tillage and straw management practices. The treatments were conventional tillage (CT), no tillage with no straw returning (NT), conventional tillage with straw returning (CTS), and no tillage with straw returning (NTS). Stable oxygen isotope (^18^O) analysis was used to determine root water uptake, evaporation (E) and transpiration (T). The results showed that wheat root absorbed water from greater depths as the growth stages advanced, and water was absorbed from deeper in the soil profile under NTS than other treatments. Transpiration changed greatly as the growth period advanced, with an initial increase, before then decreasing. The maximum transpiration occurred at jointing stage to flowering stage. Compared with CT, NTS, CTS, and NT decreased evaporation (E) and significantly increased transpiration (T) by 21.7%, 13.9%, and 7.2% in two growing seasons, respectively. Therefore, the transpiration to evapotranspiration ratio (T/ET) under NTS, CTS, and NT were 24.7%, 17.0%, and 11.1% higher than CT in two growing seasons, respectively. Our findings demonstrate that conservation tillage not only enhances root water uptake from deeper soil but also optimizes ET components by enhancing T while reducing E, thereby improving WUE in wheat. The integration of no-tillage with straw returning under NTS produced a synergistic effect that further optimized root water uptake and ET components, resulting in the greatest enhancements in both grain yield and WUE. Elucidation of this underlying physical mechanism advances the understanding of efficient water utilization in wheat under conservation tillage, thus providing insights for selecting appropriate conservation tillage in semiarid regions.

## 1 Introduction

In China, semiarid and arid dryland comprise approximately 56% of all the cultivated land, and these areas are very important for ensuring food security (Jia et al., 2018; Wu et al., 2026). Precipitation is the main water source for crop production in semiarid regions, which are dominated by dryland agriculture (Zhao et al., 2024). The interannual precipitation in these regions ranges from 250 to 600mm, and over 60% occurs during July, August, and September (Li et al., 2000; Yang et al., 2026; Zhang et al., 2019). Crop productivity and sustainable agriculture are threatened by the limited and uneven precipitation, and the grain yield and water use efficiency (WUE) are frequently reduced (Zhang et al., 2021). Therefore, the improvement of WUE is important for ensuring food security and sustainable agricultural development in semi-arid areas. In order to improve the utilization rate of rainwater, it is necessary to study the water consumption rules of crops, primarily including the root water absorption of crops and the way of field water consumption.

Root water absorption is a critical component of water circulation in Soil-Plant-Atmosphere Continuum (SPAC), it is essential to study this process to understand the principles of crop water consumption (Cai et al., 2022; Zhu et al., 2024). Stable hydrogen and oxygen isotope techniques have been found particularly effective and feasible to study plant root water uptake and widely used in various plants and regions (Corneo et al., 2018; Dawson and Ehleringer, 1991; Fu et al., 2024). This technology is based on the principle that no isotopic fractionation occurs during water absorption by plant roots and its subsequent transport to the shoots. Therefore, the plant xylem water is isotopically identical to the soil water taken up by roots (Chen et al., 2020; Jiang et al., 2022). So the water sources used by plants can be determined by comparing the isotopic composition of potential water sources with that of the xylem water (Corneo et al., 2018; Dawson et al., 2002; Ehleringer and Dawson, 1992; Miguez-Macho and Fan, 2021). Numerous studies have demonstrated a significant positive correlation and high consistency between δD and δ¹^8^O across various water samples (Liu et al., 2020; Wang et al., 2019). However, the relative mass difference between ¹^8^O and ¹^6^O is less than that between D and H, resulting in less isotopic fractionation for oxygen isotopes compared to hydrogen isotopes (Lin et al., 2026; Sharp, 2007). Consequently, δ¹^8^O has been preferentially selected for stable isotope analysis in most previous studies (Corneo et al., 2018; Xu et al., 2021).

Evapotranspiration (ET) is the principal pathway of water consumption in field ecosystems and represents a critical component of water movement within the SPAC (Orlowski et al., 2023). It comprises both evaporation (E) from soil and other surfaces and transpiration (T) through plant stomata (Morgan and Caylor, 2023). Stable hydrogen and oxygen isotope techniques also has been proven to be an efficient and non-destructive method for ET partitioning (Rothfuss et al., 2021). The underlying principle of this method lies in that evaporation causes isotopic fractionation of soil water, leading to heavier isotopes enriched in the surface soil, whereas transpiration is generally considered a non-fractionating process under isotopic steady state (ISS) (Howlider et al., 2025; Jasechko et al., 2013). In recent years, researchers have systematically elucidated the seasonal variations and environmental drivers of ET components in ecosystem such as farmland and forest by applying stable hydrogen and oxygen isotope techniques (Li et al., 2024; Matos et al., 2025; Yang et al., 2025). However, these researches were predominantly focused on different ecosystem types, climatic and environmental conditions, little studies were conducted to investigate the regulatory mechanisms of ET components under various agronomic practices.

Conservation tillage serves as a primary strategy in dryland farming systems, plays important role in addressing water scarcity in arid and semiarid regions (Du et al., 2026; Huang et al., 2008). Centered on no tillage and straw returning, it effectively preserves soil structure and increases water infiltration by minimizing soil disturbance and maintaining surface cover (Achankeng and Cornelis, 2023; Ding et al., 2023; Liu et al., 2024). Many studies have demonstrated that the conservation tillage promotes the use of limited water by crops and is highly effective for improving the WUE and crop productivity (Huang et al., 2021; Shao et al., 2016). No tillage can preserve favorable soil structure, maintain soil capillary continuity to promote water infiltration and retention, and enhance crop growth, thereby improving crop WUE (Gao et al., 2017; Liebhard et al., 2022). Straw returning can suppress the loss of soil moisture through evaporation, and also increase organic matter content to improve soil structure and water retention capacity, thereby improving crop WUE (Jiang et al., 2024; Qin et al., 2024). Different tillage practices and straw management measures influence crop WUE; therefore, investigating the enhancement mechanisms of these practices is of significant importance.

Currently, preliminary knowledge of conservation tillage in enhancing soil water content, crop yield, and WUE has been recognized (Ding et al., 2023; Huang et al., 2021; Liebhard et al., 2022). Previous studies have primarily focused on crop root morphological characteristics and total evapotranspiration, yet few have considered the patterns of root water uptake and quantified soil evaporation and plant transpiration (Li et al., 2017; Qin et al., 2024). Moreover, how to improve WUE from the perspective of root water absorption and ET components remains poorly understood. Therefore, a better understanding of the mechanism of conservation tillage drives optimization of root water uptake and ET components would be of great theoretical and practical value for selecting appropriate conservation tillage practices to improve wheat production and WUE in semi-arid areas. In the present study, a two-year field experiment was established in a semiarid region of Northwestern China to measure the stable oxygen isotope (δ^18^O) values in the different soil profiles and wheat stem. The objectives of this study were: (1) to explore the effects of different tillage and straw management practices on the wheat root water uptake characteristics; (2) to quantify ET components in order to determine the effect of different tillage and straw management practices on water consumption in wheat field; (3) to evaluate the maize yield and WUE in order to assess optimization effect of conservation tillage on wheat root water uptake and ET components to improve maize production and WUE.

## 2 Materials and methods

### 2.1 Experimental site

**Figure 1:**
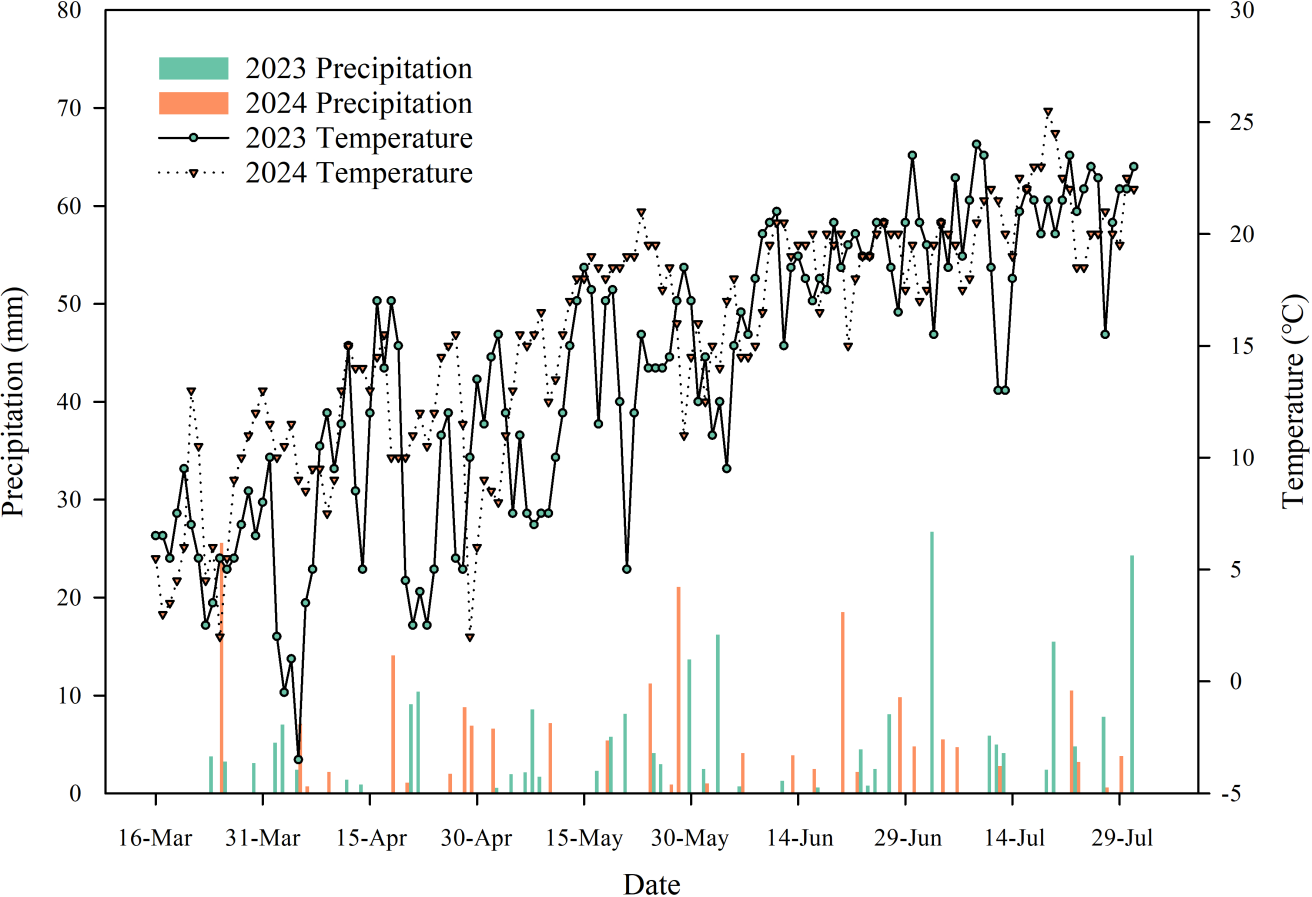
Daily precipitation and temperature during the wheat growing seasons at the experimental site in 2023 and 2024.

The field experiment was performed during 2023 and 2024 at the Rainfed Agricultural Experimental Station of Gansu Agricultural University located in Lijiabao Town, Dingxi City, Gansu province, Northwestern China (35°28′N, 104°44′E, 1971m above sea level). This site is representative of the semiarid region of the Loess Plateau and it has a warm temperate climate. Long-term average annual precipitation was 390mm and about 60% occurred during July to September. Long-term average annual cumulative air temperature >10 °C is 2240 °C and annual radiation is 5930 MJ m^−2^. The average annual air temperature was 6.5 °C and the annual accumulated sunshine hours was 2480h year^–1^. The total amounts of precipitation during the wheat growing seasons in 2023 and 2024 were 200.1mm and 180.7mm, respectively, with daily average air temperatures of 13.6°C and 15.3°C (**Figure 1**). The soil at the experimental site was determined to be Calcic Cambisol. The soil in the 0–200 cm layer had the following physical and chemical properties: bulk density of 1.27g cm^−3^, pH of 8.35g kg^–1^, total nitrogen (N) content of 0.59g kg^–1^, available phosphorus content of 12.30 mg kg^–1^, and available potassium content of 335.80 mg kg^–1^ (Du et al., 2023).

### 2.2 Experimental design and field management

The study was based on a 22 yr long-term conservation tillage experiment that initiated in 2001.A randomized block design was applied in the field experiment with the following four treatments: conventional tillage (CT), no tillage with no straw returning (NT), conventional tillage with straw returning (CTS), no tillage with straw returning (NTS). Three replicates were conducted for each treatment, and thus twelve plots were established and each covered 80 m^2^ (20 × 4m). A double sequence rotation cropping pattern involving the interannual rotation of spring wheat and peas was used. In each growing season, both wheat and pea were grown in monocropping. All measurements in this study were taken from the wheat plots.

Tillage practices for the CT, and CTS involved moldboard plowing to a depth of 20cm three times in the fall, followed by harrowing twice in the spring prior to sowing. All the straw of the previous crops (peas, for the wheat tested in this study) were retained for the CTS and NTS treatments, and the straw was incorporated into the soil at the first plough for the CTS treatment, while the straw was mulched on the soil surface for NTS treatment. In all plots, a no-till planter was used for the application of seed sowing and fertilizers at the same time. The spring wheat cultivar Dingxi 40 was sown at a rate of 187.5kg ha^-1^ with a row spacing of 20cm on March 18 in 2023 and March 22 in 2024. The pea cultivar Lvnong 2 was sown at a rate of 180kg ha^-1^ with a row spacing of 24cm on April 7 in 2023 and April 7 in 2024. All treatments were fertilized with urea and calcium superphosphate at rates of 105kg N ha^−1^ and 105kg ha^−1^ P_2_O_5_ for spring wheat and 20kg N ha^−1^ and 105kg ha^−1^ P_2_O_5_ for pea, respectively. Irrigation was not provided in both years. Weeds were eradicated manually during growing seasons and with herbicide during the fallow period according to local management practices. The spring wheat was harvested on July 25 in 2023 and July 19 in 2024 and that of pea was July 7 in 2023 and June 23 in 2024.

### 2.3 Sampling and measurements

#### 2.3.1 Stem water

Spring wheat stem base samples for root water uptake analysis were collected at jointing stage, flowering stage, and filling stage. Three representative plants were selected at about 8:00 a.m. on a sunny day. Spring wheat stem samples for ET partitioning analysis were collected on five sunny days during sowing stage to jointing stage, jointing stage to flowering stage, flowering stage to filling stage, and filling stage to maturity stage. And the stem was sampled at about 12:00~14:00 on sunny days, when the wheat under ISS. To avoid isotope fractionation caused by plant transpiration, chlorophyll-free wheat stems at 5cm above the soil surface and away from the leaves were selected. And the stem samples were put in a 12ml glass bottle, sealed with parafilm to prevent evaporation, and refrigerated immediately.

#### 2.3.2 Soil water

Soil samples for root water uptake analysis were sampled at the same time and the same place as the wheat stem samples for root water uptake analysis were collected. And the sampling time was chosen when there was no precipitation for at least 3 days to avoid excessively wet conditions of soil. Soil samples were collected using a hand-held soil ferric auger (54mm diameter) and taken to a soil depth of 200cm (0–5 cm, 5–10 cm, 10–30 cm, 30–50 cm, 50–80 cm, 80–110 cm, 110–140 cm, 140–170 cm, and 170–200 cm). After removed root the soil samples were put into a 100ml plastic bottle sealed with parafilm to prevent evaporation and refrigerated immediately for the determination of soil water isotopes.

The soil water content (SWC) was measured at sowing stage, jointing stage, flowering stage, filling stage and maturity stage. For 0−5 and 5−10 cm soil layers, the oven-drying method was employed. And the volumetric SWC was calculated by weight gravimetric SWC multiplied by corresponding soil bulk density. The volumetric SWC for 10–200 cm soil layers was measured using a time domain reflectometry soil moisture sensor (Trime-Pico IPH/T3, IMKO GmbH, Ettlingen, Germany).

Stem and soil water were extracted using the vacuum extraction system (LI-2000, LICA, China) according to Cryogenic vacuum distillation method. δ^18^O in water were measured using the TC/EA method by a Isoprime 100 mass spectrometer, which were expressed as: δ (‰)=(R_M_-R_S_)/R_S_•1000, where R_M_ and R_S_ are the^18^O/^16^O molar ratios of the sample and standard water (V-SMOW), the analytical precision was <0.1‰ for δ^18^O.

#### 2.3.3 Rain water

The rain samples were collected in a plastic bottle through a 200mm diameter funnel, in which a table tennis ball was placed to prevent the water sample in the bottle from evaporating. The rain water were gathered into air-tight glass bottles and refrigerated at 4 °C immediately after measured volume after a rain event. δ^18^O were measured as stem and soil water.

#### 2.3.4 Wheat yield

The grain yield was determined at a water content of 12% after manually harvesting the entire plot exclude the edges (0.5m). At the same time, the number of spikes were counted from three rows with a length of 5m randomly selected in each plot. And 20 representative spikes were randomly taken in each plot for determination of the grains per spike and the weight of 1,000 grains.

### 2.4 Data calculation

#### 2.4.1 Evaporation and transpiration

The partitioning of ET was based on soil water balance and isotope mass balance (Hsieh et al., 1998; Robertson and Gazis, 2006). This study was carried out in the Loess Plateau of Northwestern China. In this region, the groundwater table was located at a depth of about 80m below the surface, which resulted in the upward flow being negligible. Given the low precipitation and flat terrain of the experimental site in this semi-arid region, both deep percolation and runoff were considered negligible and therefore excluded from the analysis. Thus, the soil water balance and isotope mass balance equations are shown in Equations 1–3:

(1) $$m_{f}-m_{i}=m_{p}-m_{e}-m_{t}$$

(2) $$m_{f}\delta_{f}-m_{i}\delta_{i}=m_{p}\delta_{p}-m_{e}\delta_{e}-m_{t}\delta_{t}$$

(3) $$m_{ET}=m_{e}+m_{t}$$

Where m_f_ (mm) and m_i_ (mm) represent the soil water storage at final and initial state; m_p_ (mm) represents precipitation amount; m_e_ (mm) represents evaporation amount; m_t_ (mm) represents transpiration amount; m_ET_ (mm) represents evapotranspiration amount; δ_f_ (‰) and δ_i_ (‰) represent the δ^18^O value of soil water at final and initial state; δ_p_ (‰) represents the δ^18^O value of precipitation; δ_e_ (‰) represents the δ^18^O value of evaporation, assuming that the evaporation is carried out in isotopic equilibrium with soil water, which can be calculated based on the isotope fractionation equation α_liquid-vapor_=(δ_f_+1000)/(δ_e_+1000), the value of α is 1.0095 at 20°C, and while the α value changes with temperature, this variation has a negligible effect on the final calculated proportions (Robertson and Gazis, 2006); δ_t_ (‰) represents the δ^18^O value of transpiration, which equals the δ^18^O value of plant source water and can be measured directly by wheat stem under ISS (Xu et al., 2023).

These equations are also based on the following assumptions: lateral difference of the soil properties and lateral movement of the soil water are both minimal and ignored; the isotope value of the collected rainwater is identical to that of water immediately entered the soil; the soil particles size are fine to medium fine; and soil water movement occurs by piston infiltration (Hsieh et al., 1998).

The ratio of evaporation to evapotranspiration (F_e_, %) and transpiration to evapotranspiration (F_t_, %) were calculated according to Equations 4, 5:

(4) $$F_{e}={\text{m}}_{e}/{\text{m}}_{ET}\times 100$$

(5) $$F_{t}={\text{m}}_{t}/{\text{m}}_{ET}\times 100$$

#### 2.4.2 Water use efficiency

The water use efficiency (WUE, kg ha^-1^ mm^-1^) were calculated according to Equation 6:

(6) $$\text{WUE}=\text{Y}/m_{ET}$$

Where Y was grain yield (kg ha^-1^), m_ET_ (mm) was total ET amount during the whole growing period.

### 2.5 Statistical analysis

Analysis of variance (ANOVA) was conducted using SPSS 25.0 (IBM Inc., Armonk, NY, USA). Differences in the mean results for each treatment were calculated using the least significant difference test (LSD) at a probability level of 0.05. Analyses were conducted separately for each sampling event. All figures were prepared using SigmaPlot 16.0.

## 3 Results

### 3.1 Soil water content

**Figure 2:**
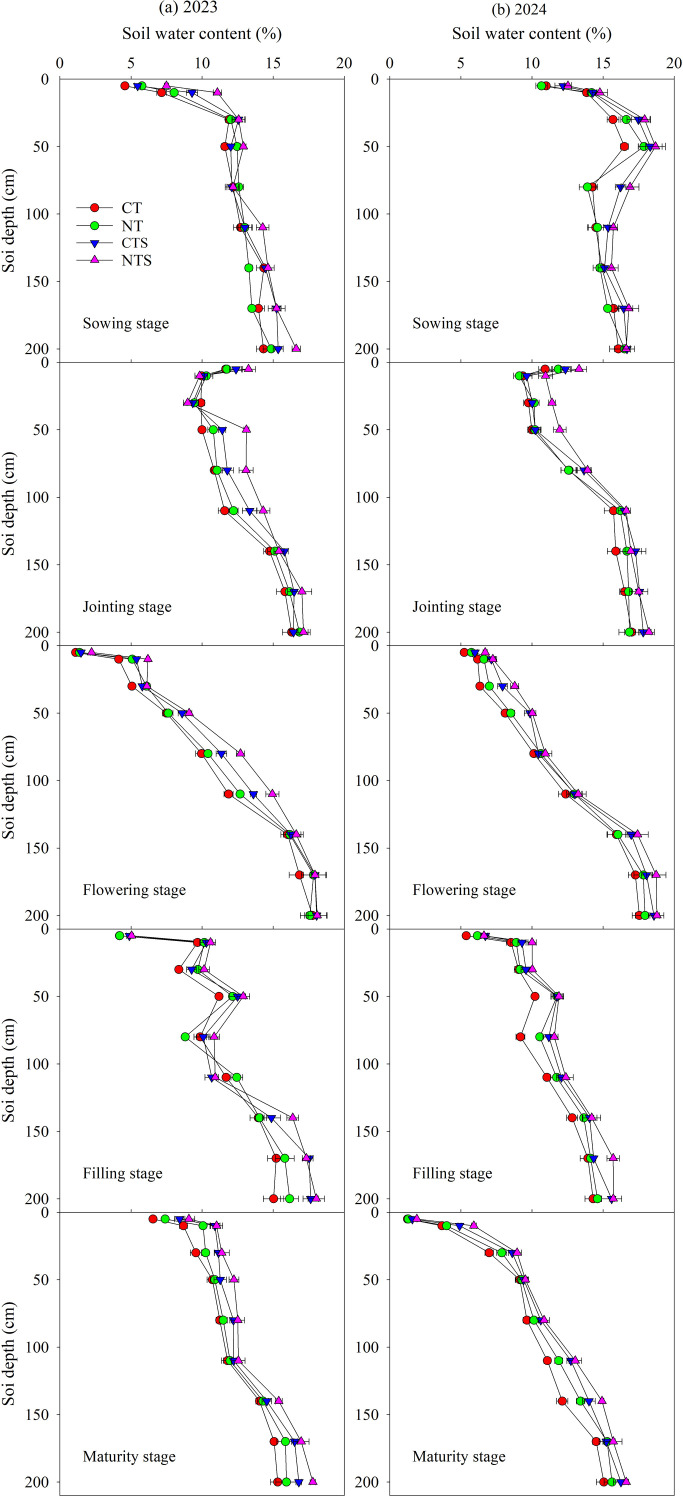
Volumetric soil water content (SWC) in the 0–200 cm soil profiles under different tillage and straw management practices during the 2023 and 2024 growing seasons. CT, conventional tillage; NT, no tillage with no straw returning; CTS, conventional tillage with straw returning; NTS, no tillage with straw returning. The vertical bars represent the standard deviation.

Tillage and straw management practices significantly affected soil moisture status, and SWC varied spatially and temporally (**Figure 2**). Both CTS and NTS were significantly higher than CT, but there were no significant differences between NT and CT during both growing seasons of 2023 and 2024. At sowing stage in 2023, CTS and NTS significantly increased SWC in 0–10 cm by 24.7% and 59.3%, respectively. At jointing stage in 2023, CTS and NTS obtained higher SWC in 50–110 cm profile, where they were 12.6% and 25.1% higher than CT, respectively. At flowering stage in 2023, CTS and NTS significantly increased SWC in 0–110 cm by 20.3% and 41.0%, respectively. At filling stage in 2023, CTS and NTS obtained higher SWC in all soil profiles except for 110cm where they were 10.9% and 15.9% higher than CT, respectively. At maturity stage in 2023, CTS and NTS obtained increases in SWC in all the soil profiles by an average of 12.3% and 17.2%, respectively. Whereas CTS and NTS obtained higher SWC in all soil profiles during all growth stages in 2024, CTS were 7.3%, 6.0%, 11.3%, 11.9%, and 15.6% higher than CT in sowing stage, jointing stage, flowing stage, filling stage, and maturity stage, respectively; NTS were 10.0%, 12.4%, 16.4%, 15.4%, and 24.2% higher than CT, respectively.

### 3.2 Wheat root water uptake

**Figure 3:**
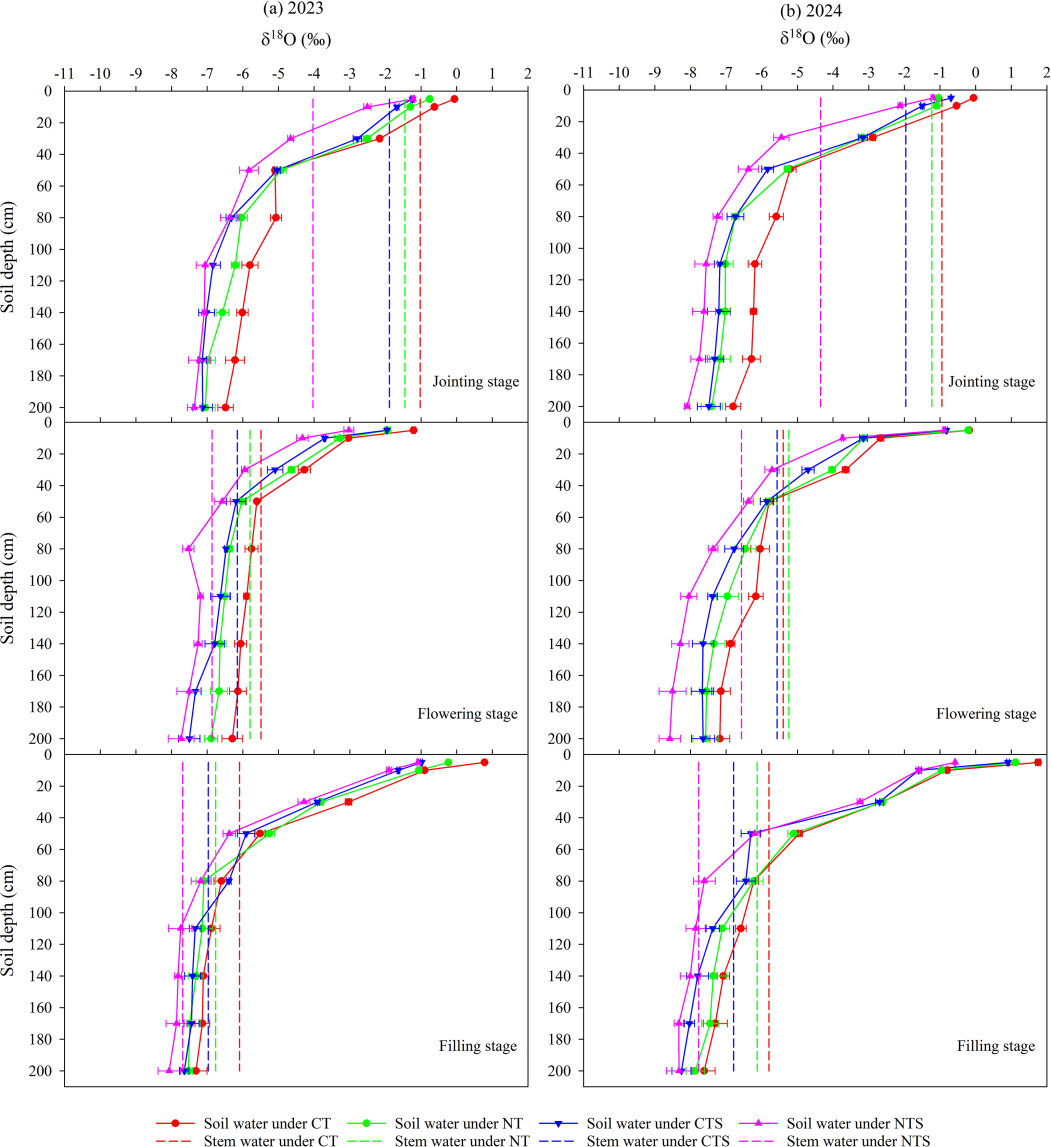
δ^18^O in soil water from 0–200 cm soil profiles and wheat stem water under different tillage and straw management practices during the 2023 and 2024 growing seasons. CT, conventional tillage; NT, no tillage with no straw returning; CTS, conventional tillage with straw returning; NTS, no tillage with straw returning. The vertical bars represent the standard deviation.

**Figure 3** presents δ^18^O value in the soil water and stem water under different treatments during 2023 and 2024 growing seasons. The oxygen isotopes in the soil water exhibited a distinct gradient distribution in the vertical profile, with the highest enrichment observed in the surface layer. Throughout both growing seasons, δ¹^8^O values fluctuated within the soil profile, showing the most significant variation in the subsoil and gradually decreasing with depth. In each growth period during the two years, the δ^18^O values under CTS and NTS differed significantly from those under CT, with more isotopic enrichment under the latter.

The depth of root water uptake is indicated in **Figure 3**, where the δ^18^O values for the stem water and soil water intersected in a specific profile, thereby demonstrating that water uptake mainly occurred from this layer. At jointing stage during the two growing seasons, the intersection of the δ^18^O value for the stem water with that for the soil water occurred in the 10–20 cm layer under CT, NT and CTS, 20–30 cm layer under NTS. At flowering stage during the two growing seasons, the intersections all occurred in the 40–50 cm layer under CT, NT, and CTS, whereas in 50–60 cm layer in NTS. At filling stage during the two growing seasons, the intersections occurred in the 60–80 cm layer under CT and NT, and in the 80–100 cm and 100–110 cm layer under CTS and NTS, respectively. Thus, the roots mainly absorbed water from the 10–20 cm layer at jointing stage and from the 40–50 cm layer at flowering stage under CT, NT, and CTS. However, at filling stage, the roots mainly absorbed water from the 60–80 cm layer under CT and NT, and from the 80–100 cm layer under CTS. The roots under NTS absorbed water from deeper soil layers compared to other treatments throughout all growth stages, with absorption depths of 20–30 cm during the jointing stage, 50–60 cm during flowering stage, and 100–110 cm during filling stage. The roots acquired water from greater depths as the growth stages advanced, and water was absorbed from deeper in the soil layer under NTS than other treatments.

### 3.3 Transpiration (T) and transpiration to evapotranspiration ratio (T/ET)

The evapotranspiration, evaporation, transpiration, E/ET and T/ET values are shown in **Tables 1** and **Table 2**. We observed that transpiration changed significantly as the growth period advanced; however, it increased initially but decreased later on. The maximum transpiration occurred at jointing stage to flowering stage. NTS significantly increased transpiration and T/ET at all growth stages in two growing seasons. At sowing stage to jointing stage, jointing stage to flowering stage, flowering stage to filling stage, and filling stage to maturity stage, transpiration under NTS were 43.2%, 13.1%, 2.6%, and 30.4% higher than CT, respectively, T/ET under NTS were 43.7%, 17.2%, 7.4%, and 35.0% higher than CT, respectively. CTS significantly increased transpiration at sowing stage to jointing stage and filling stage to maturity stage by an average of 32.1% and 27.8% in two growing seasons, respectively. And CTS significantly increased T/ET at four growth stages by an average of 33.6%, 7.4%, 6.1%, and 30.6% in two growing seasons, respectively. NT significantly increased transpiration and T/ET at sowing stage to jointing stage and filling stage to maturity stage in two growing seasons. NT increased transpiration and T/ET by an average of 19.9% and 25.1% at sowing stage to jointing stage, and increased transpiration and T/ET by an average of 26.3% and 25.4% at filling stage to maturity stage. At the whole growth stage, NTS, CTS, and NT significantly increased transpiration by an average of 21.7%, 13.9%, and 7.2% in two growing seasons, respectively, and increased T/ET by an average of 24.7%, 17.0%, and 11.1% in two growing seasons, respectively. Therefore, no tillage and straw returning increased transpiration and T/ET in two growing seasons.

**Table 1:** The evapotranspiration (ET), evaporation (E), transpiration (T), evaporation to evapotranspiration ratio (E/ET) and transpiration to evapotranspiration ratio (T/ET) under different tillage and straw management practices during the 2023 growing seasons.

Period	Treatments	ET (mm)	E (mm)	T (mm)	E/ET (%)	T/ET (%)
Sowing stage to Jointing stage	CT	74.3a	43.0a	31.3d	57.9a	42.1d
	NT	69.4b	34.6b	34.7c	49.9b	50.1c
	CTS	70.2b	31.0c	39.2b	44.1c	55.9b
	NTS	71.8ab	29.8c	41.9a	41.6d	58.4a
Jointing stage to flowing stage	CT	54.9a	16.0a	38.9bc	29.1a	70.9c
	NT	52.4b	14.9b	37.6c	28.3a	71.7c
	CTS	51.8b	12.5c	39.4b	24.1b	75.9b
	NTS	52.6b	9.2d	43.3a	17.5c	82.5a
Flowering stage to filling stage	CT	45.1a	15.7a	29.4bc	34.7a	65.3b
	NT	45.2a	14.8b	30.5b	32.6b	67.4b
	CTS	43.5a	14.6b	29.0c	33.5ab	66.5b
	NTS	44.3a	13.0c	31.3a	29.4c	70.6a
Filling stage to Maturity	CT	30.4a	14.6a	15.8c	48.0a	52.0d
	NT	30.8a	11.0b	19.8b	35.6b	64.4c
	CTS	29.9a	9.4c	20.5ab	31.4c	68.6b
	NTS	29.2a	7.9d	21.3a	26.9d	73.1a
Whole growth stage	CT	204.6a	89.2a	115.4c	43.6a	56.4d
	NT	197.8a	75.2b	122.6b	38.0b	62.0c
	CTS	195.5a	67.4c	128.1b	34.5c	65.5b
	NTS	197.8a	59.9d	137.9a	30.3d	69.7a

Values followed by different lowercase letters in a column indicate a significant difference at P < 0.05 using the LSD method. CT, conventional tillage; NT, no tillage with no straw returning; CTS, conventional tillage with straw returning; NTS, no tillage with straw returning.

**Table 2:** The evapotranspiration (ET), evaporation (E), transpiration (T), evaporation to evapotranspiration ratio (E/ET) and transpiration to evapotranspiration ratio (T/ET) under different tillage and straw management practices during the 2024 growing seasons.

Period	Treatments	ET (mm)	E (mm)	T (mm)	E/ET (%)	T/ET (%)
Sowing stage to Jointing stage	CT	119.2a	71.0a	48.2d	59.6a	40.4c
	NT	117.1a	55.1b	62.1c	47.0b	53.0b
	CTS	123.3a	56.3b	67.0b	45.7b	54.3b
	NTS	122.2a	48.7c	73.5a	39.9c	60.1a
Jointing stage to flowing stage	CT	80.5a	22.3a	58.2c	27.7a	72.3c
	NT	77.0a	20.8b	56.2c	27.0a	73.0c
	CTS	78.5a	17.3c	61.2b	22.1b	77.9b
	NTS	78.5a	11.6d	66.9a	14.7c	85.3a
Flowering stage to filling stage	CT	52.0a	17.8a	34.2a	34.2a	65.8b
	NT	47.1b	15.9b	31.3b	33.7a	66.3b
	CTS	48.0b	13.1d	34.8a	27.4c	72.6a
	NTS	48.1b	14.3c	33.8a	29.8b	70.2a
Filling stage to Maturity	CT	28.2a	14.4a	13.9b	50.8a	49.2b
	NT	28.3a	10.6b	17.7a	37.5b	62.5a
	CTS	27.5a	10.0c	17.5a	36.4b	63.6a
	NTS	27.4a	10.0c	17.5a	36.3b	63.7a
Whole growth stage	CT	280.0a	125.4a	154.6d	44.8a	55.2d
	NT	269.6a	102.3b	167.3c	38.0b	62.0c
	CTS	277.2a	96.7c	180.5b	34.9c	65.1b
	NTS	276.3a	84.6d	191.6a	30.6d	69.4a

Values followed by different lowercase letters in a column indicate a significant difference at P < 0.05 using the LSD method. CT, conventional tillage; NT, no tillage with no straw returning; CTS, conventional tillage with straw returning; NTS, no tillage with straw returning.

### 3.4 Wheat yield and water use efficiency

Tillage and straw management practices affected grain yield and its components during both growing seasons (**Table 3**). Compared with CT, conservation tillage increased spikes per unit area, with NT, CTS, and NTS by an average of 12.6%, 16.9%, and 27.3%, respectively. The effect of conservation tillage on grains per spike and grain yield varied between the two years. In 2023, there were no significant differences between NT and CTS and CT, while NTS significantly increased grains per spike by 15.1% compared to CT. However, in 2024, in comparison to CT grains per spike, recorded a significant increase with NT, CTS and NTS by 15.2%, 33.3% and 41.2%, respectively. There were no significant differences between conservation tillage and CT in 2023, while NTS significantly increased 1000-grains weight by 7.6% in 2024. Conservation tillage significantly increased the grain yield and WUE during both growing seasons. NT, CTS, and NTS increased grain yield by an average of 13.8%, 27.8%, and 50.0%, and that of WUE were 21.7%, 28.0%, and 53.7% during both the growing season over conventional tillage, respectively. Moreover, the grain yield and WUE under NTS were significantly higher than NT and CTS.

**Table 3:** Wheat yield and water use efficiency (WUE) under different tillage and straw management practices during the 2023 and 2024 growing seasons.

Year	Treatments	Spike (No. m^-2^)	Grains per spike (No.)	1000−grain weight (g)	Grain yield (kg ha^−1^)	WUE (kg ha^−1^ mm^−1^)
2023	CT	291.3c	31.8b	31.4ab	713.8d	3.5d
	NT	318.4b	32.6b	30.5b	868.8c	4.4c
	CTS	329.0b	32.4b	31.5ab	1016.4b	5.2b
	NTS	412.4a	36.6a	32.9a	1174.6a	5.9a
2024	CT	350.0c	26.1d	32.7b	1452.2d	5.2d
	NT	405.4ab	30.1c	33.5b	1537.5c	6.1b
	CTS	422.9a	34.8b	33.4b	1643.7b	5.5c
	NTS	395.5b	36.9a	35.2a	1966.5a	7.1a

Values followed by different lowercase letters in a column indicate a significant difference at *P* < 0.05 using the LSD method. CT, conventional tillage; NT, no tillage with no straw returning; CTS, conventional tillage with straw returning; NTS, no tillage with straw returning. WUE, water use efficiency.

## 4 Discussion

### 4.1 The effect of conservation tillage on soil water content

Conservation tillage, like no tillage, straw returning, or in combination, improves soil structure by increasing porosity and aggregate stability. These enhancements promote soil aeration and water retention, ultimately resulting in a more favorable soil water distribution (Gao et al., 2017; Pečan et al., 2023). Overall, SWC in surface soil typically exhibits greater fluctuations than in deeper layers (**Figure 2**). This is because the surface soil is in direct contact with the atmosphere, responding more rapidly to evaporation and precipitation, whereas the influence of these processes on deeper soil is relatively delayed (Qing et al., 2023; Tao et al., 2022). During the sowing stage, the positive effects of CTS and NTS were most evident in the shallow 0–10 cm layer (**Figure 2**). This is because straw returning can reduce nonproductive evaporation from the topsoil, thereby effectively conserving soil moisture from the fallow period and consequently increasing the soil water storage in the early sowing stage (Dong et al., 2022; Wang et al., 2023). This enhanced water reserve is crucial for ensuring successful seed germination and seedling establishment, laying a foundation for wheat growth and subsequent yield enhancement (Alemie et al., 2026; Deng et al., 2003). The moisture conservation capacity of CTS and NTS during the early growth stage is of critical importance in rain-fed agricultural systems characterized by frequent spring drought conditions (Sadiq et al., 2021). As the growing stage advanced, the benefits of conservation tillage extended into deeper soil layers, indicating that such practices not only conserve surface moisture but also promote deeper water infiltration, thereby establishing a more reliable reservoir of soil moisture within the root zone to sustain crops during critical reproductive growth phases (Lu et al., 2024; Zhang et al., 2022). The higher SWC in all soil profiles during all growth stages under CTS and NTS in 2024 may result from more precipitation compared with 2023 (**Figure 1**).

A particularly important finding in this study is that straw returning consistently exhibited significantly higher SWC (**Figure 2**). This observation is consistent with findings of numerous previous studies (Jaćimović et al., 2023; Li et al., 2020). The underlying mechanism can be largely ascribed to the physical barrier formed by straw returning, which effectively inhibits evaporation losses from the soil surface (Wang et al., 2023). Moreover, the progressive decomposition of straw amendments enhances soil structure, elevates organic matter content, and facilitates the formation of water-stable aggregates, collectively contributing to improved soil water-holding capacity and infiltration rates (Page et al., 2019; Sekaran et al., 2021). Straw returning is a key measure for improving soil water retention capacity, and when combined with no-till practices, it produces a better synergistic effect. In marked contrast, the absence of a significant difference in SWC between NT and CT indicates no tillage without straw returning has no obvious advantages in terms of moisture retention. This strongly suggests that straw returning plays a more influential role in soil water retention than the mere reduction of mechanical disturbance. Another notable result was the consistent superiority of the NTS treatment over CTS (**Figure 2**). Du et al. (2023) also found that no tillage exhibited higher SWC and WUE than conventional tillage under the condition of straw returning. The enhanced performance under NTS is likely attributed to the complete minimization of soil disturbance, which preserves the integrity and protective function of the surface mulch layer. In contrast, CTS turns straw into soil while ploughing that may increase evaporation by exposing moist subsoil to the atmosphere and disrupting soil capillary continuity, thereby moderately reducing its overall water conservation efficiency relative to NTS.

### 4.2 Transpiration and yield

The dynamics of transpiration across growth stages followed a consistent pattern, characterized by an initial increase culminating during the jointing to flowering stages, followed by a gradual decline toward maturity (**Tables 1**, **2**). This pattern was closely related to the phenological development of the crop. Previous studies have shown that conservation tillage can significantly suppress evaporation and enhance transpiration (Deng et al., 2006; Jiang et al., 2026). Similar results were obtained in our study. Throughout the wheat growing season, conservation tillage practices, compared to CT, reduced non-productive soil evaporation (**Tables 1**, **2**). This thereby enhanced SWC, which subsequently promoted wheat growth and development, and consequently led to increased physiological transpiration (Wang et al., 2024; Xu et al., 2023). Simultaneously, the reduction in evaporation under conservation tillage also accounts for the less enriched values of δ¹^8^O observed in these treatments compared to CT (**Figure 3**). Furthermore, significantly lower SWC and a more enriched surface soil δ¹^8^O value in NT compared to CTS and NTS observed in this study partly due to the enhanced soil evaporation resulting from the absence of a straw mulch layer (Zhang et al., 2025). Conservation tillage significantly increased wheat transpiration at every growth stage, with the most substantial improvements observed during the early (sowing to jointing) and late (filling to maturity) stages (**Tables 1**, **2**). This indicates that the positive effects of conservation tillage are more pronounced during the initial and final phases of crop development. No tillage promotes transpiration can be attributed to reduced soil disturbance, which maintains favorable soil structure and thereby promotes crop growth and development (Galdos et al., 2019; Liebhard et al., 2022). Straw returning contributes to higher transpiration by not only suppressing evaporation to ensure adequate water availability but also by enhancing soil organic matter (Jin et al., 2020). This improvement in organic matter optimizes soil physicochemical properties, consequently ensuring nutrient supply for crops (Dass et al., 2025; Martínez et al., 2013; Sellami and Lavini, 2023). Furthermore, it effectively regulates the soil micro-environment, thereby creating a more favorable overall growth habitat (Zhang et al., 2024). These combined effects ultimately promote crop growth and development, leading to increased transpiration. The synergistic interaction between no tillage and straw returning leads to a superior enhancement of crop transpiration (Li et al., 2023; Peng et al., 2019). This effect is clearly demonstrated in the present study by the higher transpiration observed under NTS compared to the NT and CTS treatments.

Transpiration is closely related to crop yield. A study by Xu et al. (2023) indicated that the ridge-furrow mulching system promoted transpiration at all growth stages, thereby increasing dry matter accumulation and yield. In this study, conservation tillage significantly increased wheat transpiration (**Tables 1**, **2**), which contributed to high grain yield (**Table 3**). Conservation tillage has been widely recognized as an effective strategy for accelerating crop growth and development due to improved soil physical, chemical, and biological properties (Page et al., 2020; Yuan et al., 2023). The increase in spike number per unit area under all conservation tillage treatments, and particularly under NTS, may be due to improved early growth conditions during the early growth stage facilitate seedling establishment (Djouadi et al., 2021). The favorable development of seedlings also contributes to the significant increase in transpiration during the early growth stage of wheat. Annual variations in grains per spike and 1000-grain weight may arise from climatic influences on water availability during critical growth stages, particularly the spikelet differentiation and grain filling periods, ultimately affecting the expression of yield components. The significant and consistent increase in grain yield under conservation tillage across seasons, with NTS demonstrating superiority over all other treatments, further validates the critical role of straw returning coupled with no tillage in stabilizing and boosting crop production.

### 4.3 Wheat root water uptake, transpiration to evapotranspiration ratio and water use efficiency

We found that wheat roots absorbed water from deeper soil as the growth stages advanced according to stable hydrogen and oxygen isotopes technology (**Figure 3**), and this was consistent with the root development trend during the growth period (Zhao et al., 2018). Li et al. (2011) also obtained similar results and showed that the roots absorbed water from greater soil depths as the roots developed throughout the whole growth period. However, many studies have shown that grass and herbs tend to continuously absorb water from the shallow soil layer throughout the whole growth stage, whereas trees and shrubs mainly obtain water from deeper soil layers (McCole and Stern, 2007; Prechsl et al., 2015; West et al., 2012). These different water uptake patterns may be due to various plant species possessing different root types. Our previous study of the water uptake mechanism by the roots in spring maize detected significant positive correlations between water uptake and the growth and distribution of the roots (Xu et al., 2021). In the present study, different tillage and straw management practices significantly affected the uptake of water by wheat roots, where water was absorbed from deeper soil under NTS than other treatments at all growth stages (**Figure 3**). This may be attributed to different root distributions under various tillage and straw management practices, with wheat roots in the NTS treatment extending into deeper soil layers (Du et al., 2023; Xu et al., 2021). The deeper root water uptake under CTS than CT during the critical grain filling period is attributed to its capacity to preserve soil moisture in deeper layers when the surface soil dries out due to high evaporation. Simultaneously, the improved soil structure and favorable hydrothermal conditions of straw returning promote root development, thereby enhancing access to deeper water reserves (Zhang et al., 2026). Throughout all growth stages, wheat roots under the NTS treatment absorbed water from deeper soil layers compared to other treatments (**Figure 3**). This may result from the synergistic effects of no tillage and straw returning. No tillage practices promote stable biological pores and improved soil structure, encouraging deeper root penetration (Duchene et al., 2024; Hobson et al., 2022). Meanwhile, straw returning likely prevents topsoil compaction through moisture retention, enabling roots to maintain functional activity while exploring stable water sources at greater depths (Wei et al., 2025).

The pattern of wheat root water uptake depth increasing with the progression of the growing season also represents an adaptive strategy to meet the continuously rising transpiration demand (Ma et al., 2018). Conservation tillage practices, particularly NTS, significantly increased the T/ET, indicating a fundamental shift in soil moisture loss pathways from nonproductive soil evaporation to productive plant transpiration (Tan et al., 2022). By accessing deeper soil water, wheat can maintain higher transpiration rates during critical reproductive stages, as evidenced of conservation tillage have higher T/ET than CT during the filling to maturity stage (Yang et al., 2017). Meanwhile, straw returning directly suppresses evaporation, thereby increasing the proportion of available water for transpiration (Chen et al., 2007; Wang et al., 2025). This explains why NT alone also increased T/ET, but the combination with straw (NTS and CTS) produced a more pronounced and consistent effect across the entire growing season. The data convincingly show that the integration of no-tillage and straw returning is the most effective strategy to maximize the productive component of water loss from agroecosystems.

Conservation tillage optimizes the water uptake and transpiration structure of wheat roots, ultimately enhancing the crop’s WUE. The ultimate agronomic benefit of these physiological and hydrological adjustments is reflected in the dramatically enhanced WUE. The WUE of wheat under different conservation tillage practices (NTS > CTS > NT > CT) are perfectly consistent with their capacity to promote deeper water uptake and a higher T/ET. In addition, the wheat under conservation tillage produced a higher yield using an amount of water similar to that of conventional tillage. The improvement in wheat WUE under conservation tillage can be attributed to two factors. Firstly, the reduction in unproductive soil evaporation via no tillage and straw returning. Second, and equally important, the enhanced capacity for water acquisition and transpiration supports greater yield production per unit of water consumed. The greatest improvement of WUE under NTS fully demonstrates the synergistic effect generated by the combination of no tillage and straw returning.

## 5 Conclusion

Based on a 22-year experiment in semiarid region in northwestern China, it is concluded that conservation tillage maintained a more favorable soil moisture status compared to CT. No tillage along with straw returning (NTS) enhanced root growth to optimize the water use strategy in wheat and increased water absorption from the deeper soil profile. In addition, conservation tillage not only increased T and T/ET, but also contributed substantially to yield formation through improved yield components. The synergistic interaction between no tillage and straw returning leads to a superior enhancement of crop transpiration, T/ET, grain yield and WUE. These findings suggest that conservation tillage optimizes wheat root water uptake and ET components, thereby facilitates the improvement of wheat production and WUE in semiarid regions. Consequently, NTS can be recommended as a highly effective management strategy in semiarid regions. However, long-term straw returning may may lead to an increase in the incidence of soil-borne diseases. Therefore, future research should focus on long-term monitoring of allelochemical accumulation to ensure sustainable application of NTS.

## Data Availability

The raw data supporting the conclusions of this article will be made available by the authors, without undue reservation.
